# Quantitative proteomics by iTRAQ-PRM based reveals the new characterization for gout

**DOI:** 10.1186/s12953-021-00180-0

**Published:** 2021-10-11

**Authors:** Guangqi Chen, Jiafen Cheng, Hanjie Yu, Xiao Huang, Hui Bao, Ling Qin, Ling Wang, Yaxiang Song, Xinying Liu, Ai Peng

**Affiliations:** Center for Nephrology and Clinical Metabolomics and Division of Nephrology and Rheumatology, Shanghai Tenth People’s Hospital, Tongji University School of Medicine, Shanghai, PR China

**Keywords:** Gout, iTRAQ, PRM, Histone H2A, Histone H2B, THBS1

## Abstract

**Background:**

Gout is a common and complex form of immunoreactive arthritis based on hyperuricemia, while the symptoms would turn to remission or even got worse. So, it is hard to early identify whether an asymptomatic hyperuricemia (AHU) patient will be susceptible to get acute gout attack and it is also hard to predict the process of gout remission to flare. Here, we report that the plasma proteins profile can distinguish among acute gout (AG), remission of gout (RG), AHU patients, and healthy controls.

**Methods:**

We established an isobaric tags for relative and absolute quantification (iTRAQ) and parallel reaction monitoring (PRM) based method to measure the plasma proteins for AG group (*n* = 8), RG group (*n* = 7), AHU group (*n* = 7) and healthy controls (*n* = 8).

**Results:**

Eleven differentially expressed proteins such as Histone H2A, Histone H2B, Thrombospondin-1 (THBS1), Myeloperoxidase (MPO), Complement C2, Complement component C8 beta chain (C8B), Alpha-1-acid glycoprotein 1 (ORM1), Inter-alpha-trypsin inhibitor heavy chain H4 (ITIH4), Carbonic anhydrase 1 (CA1), Serum albumin (ALB) and Multimerin-1 (MMRN1) were identified. Histone H2A, Histone H2B and THBS1 might be the strongest influential regulator to maintain the balance and stability of the gout process. The complement and coagulation cascades is one of the main functional pathways in the mechanism of gout process.

**Conclusions:**

Histone H2A, Histone H2B and THBS1 are potential candidate genes for novel biomarkers in discriminating gout attack from AHU or RG, providing new theoretical insights for the prognosis, treatment, and management of gout process.

**Trial registration:**

This study is not a clinical trial.

**Supplementary Information:**

The online version contains supplementary material available at 10.1186/s12953-021-00180-0.

## Introduction

Gout is an inflammatory arthritis caused by the deposition of monosodium urate (MSU) crystals in the joints, accompanied by severe pain, which has increasingly affected human health and reduced the living standard [1, 2]. Traditionally, there is a potential relationship between the occurrence of gout and the increase of uric acid (UA) in blood. A number of epidemiological have demonstrated recently that the incidence of hyperuricemia in Chinese main land is 13.3%, while the gout is 1.1%, and the trend is still on the rise synchronously [3, 4]. Gout attacks not only can destroy joint tissues progressively, but also result in a few comorbidities such as chronic kidney disease, cardiovascular disease and metabolic syndrome [5].

Clinically, serum UA levels in many patients with acute gout (AG) are similar to those in asymptomatic hyperuricemia (AHU), about 7% of patients with AG automatically enter to remission period, so it is extremely tough to predict the acute attack of gout by serum level of UA [5]. So, exploring differentially expressed key proteins associated with gout attacks can help us identify the different processes of gout as early as possible. However, given the wide variety of proteins in human blood, how to screen these proteins with high-throughput precision is a major challenge.

With the emergence of proteomics technology, isobaric tags for relative and absolute quantification (iTRAQ), as one of the most sensitive proteomic quantification tools, has attracted extensive attention as a research hotspot to explore the pathogenesis of diseases and predict biomarkers [6–8]. However, this proteomics doesn’t guarantee identification of specific core protein [9]. Parallel reaction monitoring (PRM) is a novel mass spectrometry method which can distinguish between interference information and real signals and has the greater selectivity to detect target protein [10]. Surprisingly, there is a paucity of iTRAQ and PRM-based literature describing the impact of expressed proteins on gout process.

Therefore, in this study we attempt to find a number of up-regulated and down-regulated proteins which can well distinguish among healthy control, AG, remission of gout (RG) and AHU based on iTRAQ. Moreover, we plan to identify proteins functions which may be involved in the pathogenesis of gout by performing Gene Ontology functional enrichment analysis and genome encyclopedia (KEGG) pathway enrichment analysis. Then we will focus on some interesting proteins to further validate by PRM.

## Materials and methods

### Blood sample collection

This study was approved by the Ethics Committee of Shanghai Tenth People’s Hospital. Eligibility criteria required healthy volunteers to have received: (1) aged between 18 and 60 years; (2) The serum UA levels were lower than 420 μmol/L for a man and 360 μmol/L for a woman; (3) without any clinically diagnostic severe diseases including but not limited to a tumor, cardiovascular, renal, nervous, digestive and mental disorders. Eligibility criteria required AHU included: (1) serum levels of UA were both greater than 420 μmol/L for a man and 360 μmol/L for a woman; (2) without self-reported history of the acute gout; (3) without receiving medical treatment; (4) without any other diseases as mentioned above. The primary patients with AG were diagnosed in accordance with the ACR/EULAR gout classification criteria in 2015 [11]. The diagnosis of RG was based on the Provisional Definition of Remission in 2016 [12]. Informed consent Ethical approval was obtained from all participants.

The plasma samples used in this study were the remaining samples of those who were clinically diagnosed in the Department of Nephrology and Rheumatology of this hospital as patients with AG, RG and AHU. The samples were collected for the clinical laboratory test with ethylenediaminetetraacetic acid (EDTA) between January 2018 and December 2019. The collected blood samples were centrifuged at 1500 g for 10 min under room temperature within 1 h after collection for the separation of plasma. After separation, the plasma samples from each participant were stored at 4 °C temporarily till transfer. The remnant plasma was transferred into a clean Eppendorf tube within 3 h after plasma separation and immediately stored at − 80 °C until analysis.

### Protein sample preparation

Forty microliters serum of each sample was acquired and diluted with 10X Binding Buffer and water. Albumin and immunoglobulin G were removed from serum samples using the ProteoExtract™ Albumin/IgG Removal Kit. Then the protein samples were re-dissolved with 250 μL SDS lysis buffer and centrifuged at 12000 g for 15 min to remove insoluble particles (repeat once). Protein concentration was determined by Bradford assay and aliquoted to store at − 80 °C. The 10 μg proteins of each sample were acquired and separated by 12% SDS-PAGE gel. Then the separation gel stained by CCB was scanned by ImageScanner (GE Healthcare, USA) at the resolution of 300dpi. Individual 100 μg protein extraction (equilibrated to 30 μL by lysis buffer) was subjected with 120 μL reducing buffer (10 mM DTT, 8 M Urea, 100 mM TEAB, pH 8.0) on 10KDa ultrafiltration tube. Iiodoacetamide was added to the final concentration of 50 mM and reacted at room temperature for 40 min. The filters were then washed twice using 100 μL dissolution buffer (300 mM TEAB), and then being centrifuged twice at 12000×g for 20 min. After removing urea, proteins were digested with sequence-grade modified trypsin. The digested peptides were desalted by C18-Reverse-Phase SPE Column.

### Proteomic analysis by iTRAQ

The peptide mixture was labeled using iTRAQ reagent 8Plex Assay Kit according to the manufacturer’s instructions (AB Sciex, USA). The iTRAQ labeled peptides were fractionated by high-pH separation using Agilent 1260 infinity II HPLC system (buffer A:10 mM HCOONH_4_, 5% ACN, pH 10.0; buffer B: 10 mM HCOONH_4_, 85% ACN, pH 10.0). The dried peptide mixture then loaded onto a column. The peptides were eluted at a flow rate of 1 ml/min with a linear gradient of 0% buffer B for 25 min, 0–7% buffer B for 25–30 min, 7–40% buffer B for 30–65 min, 40–100% buffer B for 65–70 min, 100% buffer B for 70–85 min. The elution was monitored with absorbance at 214 nm, and fractions were collected every 1 min. The fractions were resuspended and separated by nanoEasy nLC. The column was balanced with 100% buffer A (0.1% formic acid) and the peptide mixture were separated from the automatic sampler to the reversed-phase analytical column (Thermo scientific, claim PepMap RSLC 50um X 15 cm, nano viper, P/N164943) and separated with a linear gradient of buffer B (80% acetonitrile) at a flow rate of 300 nl/min. The samples separated by chromatography were further performed to LC–MS/MS analysis on a Q Exactive HF Mass spectrometer. All LC–MS/MS samples were analyzed using Mascot 2.5 software and Proteome Discoverer2.1 for protein identification and quantitative analysis. Spectral data were searched against a concatenated human reference library (https://www.uniprot.org/; accessed 5 December 2016) using Proteome Discoverer 2.1, the following parameters were set: oxidized methionine (M), Acetyl (Protein N-term) and deamidation (NQ) were selected as variable modifications, and carbamidomethyl (C) as static modifications; precursor mass tolerance 20 ppm; fragment mass tolerance 0.1 Da. Trypsin was specified as the enzyme, with 2 missed cleavages permitted. The protein screening criteria for identification were accepted if they could achieve a false discovery rate (FDR) less than 1% and differentially expressed protein were screened with fold-change, 1.2 times; *p* < 0.05. The process of Gene Ontology (GO) annotation for target proteins was carried out using Blast2GO. At first, all protein sequences were aligned to *Homo sapiens* (see project report) database downloaded from NCBI (ncbi-blast-2.2.28 + −win32.exe), only the sequences in top 10 and E-value<=1e-3 were kept. Secondly, select the GO term (database version: go_201504.obo) of the sequence with top Bit-Score by Blast2GO. Then, completed the annotation from GO terms to proteins by Blast2GO Command Line. After the elementary annotation, InterProScan were used to search EBI database by motif and then add the functional information of motif to proteins to improve annotation. Then further improvement of annotation and connection between GO terms were carried out by ANNEX. Fisher’s Exact Test were used to enrich GO terms by comparing the number of DEPs and total proteins correlated to GO terms. Pathway analysis was performed using KEGG database. Fisher’s Exact Test were used to identify the significantly enriched pathways by comparing the number of DEPs and total proteins correlated to pathways. KAAS (KEGG Automatic Annotation Server) software is used to annotate the Kyoto Encyclopedia of Genes and Genomes (KEGG) pathway of the target protein collection.

### Protein validation by PRM analysis

The sample mix was fractionated on an Agilent 1100 liquid chromatograph at pH 10. Finally, 10 fractions were collected, and run in DDA mode to obtain the protein and peptide list which were used to set up a scheduled PRM assay. A list of peptides from DDA analysis was prepared for PRM validation (at least 2 peptides per protein). Samples were loaded onto a precolumn (100 μm × 3 cm, C18, 3 μm, 150 Å) and separated on an analytical column (75 μm × 50 cm, C18, 3 μm, 120 Å) at a flow rate of 5 μl/min (buffer A: 2% ACN, 0.1% formic acid, buffer B: 95% ACN, 0.1% formic acid). A 90 min gradient was performed as follow: 0 ~ 60 min, 8-25% B; 60 ~ 79 min, 25-45% B; 79 ~ 80 min, 45-100% B; 80 ~ 90 min, 100%B. Peptides were transferred to the gaseous phase with positive ion electrospray ionization at 2.1 kV. For DDA, the top 10 precursors were acquired between 350 and 1650 m/z, dynamic exclusion of 40 s, normalized collision energy (NCE) of 27. Resolution for MS1 was 120,000, 30,000 for MS2. For PRM, precursors were targeted in a 1.2 m/z isolation window around the m/z of interest. Precursors were fragmented in HCD mode with NCE energy of 32. MS/MS was performed at 15000 resolution, an AGC target of 1e5 and a maximum injection time was 200 ms. Spectra were analyzed using Skyline2 with manual validation [13]. The software was used for retention time alignment, peak detection of peptide fragments and their quantification. The list of the peptides followed by PRM is given in the supplementary information.

### Statistical analysis

All statistical data were performed with GraphPad software (Prism 5, version 5.01; GraphPad software, Inc., San Diego, Calif), and the results of the quantitative data are presented as the mean ± SEM. The data were analyzed by one-way ANOVA test. A value of *p* < 0.05 was considered to indicate statistically significant (*, *p* < 0.05; **, *p* < 0.01; ***, *p* < 0.001).

## Result

### General characteristic

As shown in Table 1, the proportion of hypertension group of AG, RG and AHU was more than the group of CTL. Comparative analysis suggested that the variables of BUN, TC, TG, ALT, AST and LDL-C had no statistical difference, while variables of UA, SCr, HDL-C were significantly different among these four groups (*p* < 0.05).

**Table 1:** Baseline characteristics of identified subjects

Items	Control (*N* = 8)	Acute Gout (*N* = 8)	Gout Remission (*N* = 7)	Asymptomatic Hyperuricemia (*N* = 7)
Male(n)	2	8	6	6
Age (year)	28.38 ± 1.97	50.13 ± 3.92*	44.57 ± 7.19	38.57 ± 6.34
Serum UA (μmol/L)	273.0 ± 19.90	550.3 ± 45.17***	498.1 ± 55.78***	509.2 ± 19.53***
SCr (μmol/L)	59.86 ± 5.60	86.44 ± 6.92	106.8 ± 19.84*	107.5 ± 12.09*
BUN (mmol/L)	4.49 ± 0.24	4.80 ± 0.41	5.40 ± 1.14	5.93 ± 0.53
TC (mmol/L)	4.81 ± 0.13	4.21 ± 0.31	4.00 ± 0.21	4.64 ± 0.39
TG (mmol/L)	0.74 ± 0.05	1.84 ± 0.37	1.80 ± 0.22	2.33 ± 0.67*
HDL-C (mmol/L)	1.80 ± 0.10	0.95 ± 0.075***	0.98 ± 0.08***	1.18 ± 0.14***
LDL-C (mmol/L)	2.42 ± 0.08	2.42 ± 0.25	2.28 ± 0.26	2.64 ± 0.38
Hypertension(n)	0	3	3	2

Data present mean ± SD (minimum–maximum)

*UA* Uric acid, *SCr* The serum level of creatinine, *BUN* Blood urea nitrogen, *TC* Total cholesterol, *TG* Total glycerides, *HDL-C* High-density lipoprotein cholesterol, *LDL-C* Low-density lipoprotein cholesterol

*P* values were calculated by using χ2 test for gender, hypertension and one-way ANOVA test for others (*, *p* < 0.05; **, *p* < 0.01; ***, *p* < 0.001)

### Proteomic differences detected by iTRAQ

#### Identify differentially expressed proteins (DEPs)

**Fig. 1:**
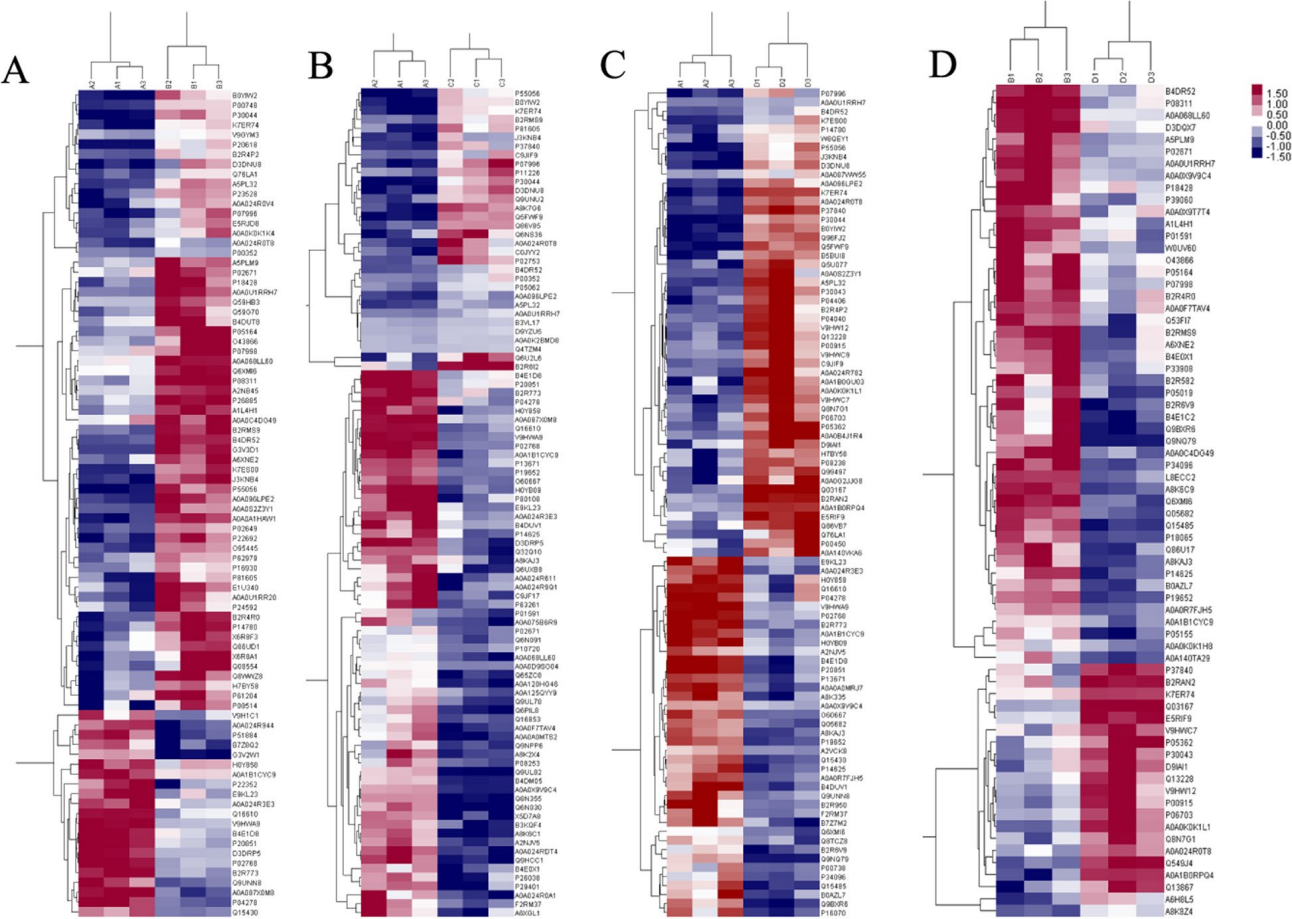
Cluster heat map of proteomics profiles in four comparison groups. Each row in the figure represents a protein, each column represents a set of samples. Regions of red or blue indicates that the differentially expressed protein is increased or decreased, respectively. White part represents there was no change in protein expression. **A** Cluster heat map of proteomics profiles in AG vs. CTL. **B** Cluster heat map of proteomics profiles in RG vs. CTL. **C** Cluster heat map of proteomics profiles in AHU vs. CTL. **D** Cluster heat map of proteomics profiles in AHU vs. AG

By iTRAQ proteomic analysis, a total of 9876 with unique peptides or polypeptide segments corresponding to 947 proteins were identified among AG, RG, AHU patients, and healthy controls (Table S1). Compared with CTL, we totally found 84 DEPs in the AG group, of which 63 proteins were up-regulated and 21 proteins were down-regulated. Compared with the CTL, we totally found 94 DEPs in the RG group, of which 32 proteins were up -regulated and 62 proteins were down-regulated. Compared with the CTL, in AHU group, we totally found 92 DEPs, of which 52 proteins were up-regulated and 40 proteins were down-regulated. Compared with the AG, in the AHU, we totally found 69 DEPs, of which 21 proteins were up-regulated and 48 proteins were down-regulated. The differential proteins in the clustering heat map were shown in Fig. 1.

#### Gene ontology (GO) functional annotation analysis

**Fig. 2:**
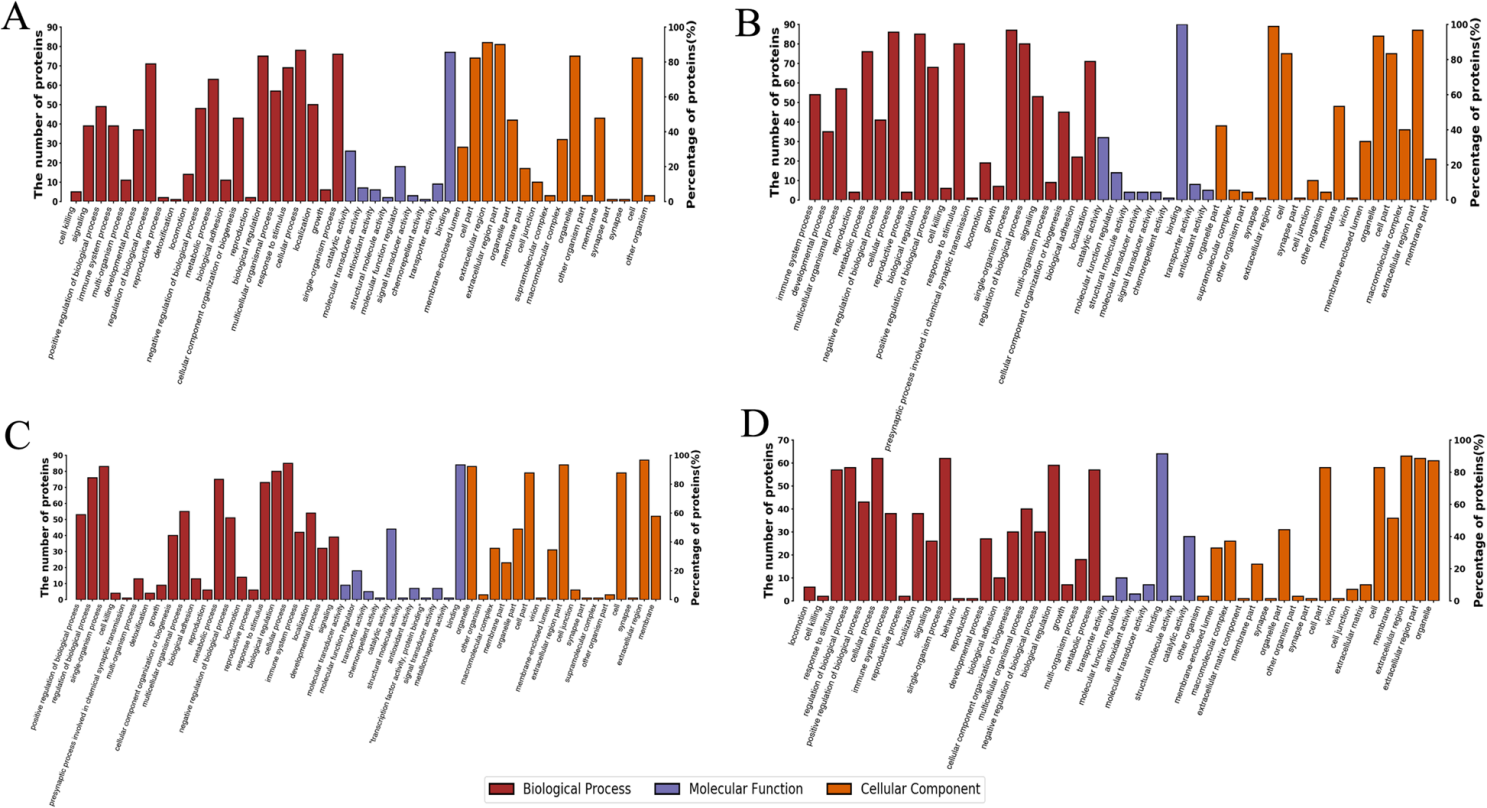
The GO annotation results of DEPs in four comparison groups. The abscissa represents the GO Level 2 explanatory information, including biological process (red), molecular function (purple) and cellular component (orange). The ordinate (right) is a representation of the number of DEPs under each functional classification, and the ordinate (left) represents the percentage of DEPs under each functional classification in the total number of DEPs. **A** The GO annotation results of DEPs in AG vs. CTL. **B** The GO annotation results of DEPs in RG vs. CTL. **C** The GO annotation results of DEPs in AHU vs. CTL. **D** The GO annotation results of DEPs in AHU vs. AG

To analyze the associated functions of the proteomics profiles in four groups, the DEPs underwent GO functional annotation based on Blast2GO software. Using Fisher’s exact test method, result of GO functional enrichment analysis could reveal the main biological processes (BP), cellular components (CC), and molecular function (MF) involved in DEPs in different groups (Fig. 2). According to the GO analysis, we found that in terms of biological processes, these proteins were mainly involved in lipid metabolism, endocytosis, vesicle-mediated transport, receptor-mediated endocytosis, anion transport, negative regulation of proteolysis, negative regulation of cell metabolic process, and negative regulation of catalytic activity. In terms of cell localization, these proteins were mainly located in extracellular space, blood microparticles, high-density lipoprotein particles, triglyceride-rich lipoprotein particles, and low-density lipoprotein particles. Significant changes occurred in some molecular functions like binding of lipid substances, lipid transport activity and peroxidase activity.

#### Kyoto encyclopedia of genes and genomes (KEGG) analysis of DEPs

**Fig. 3:**
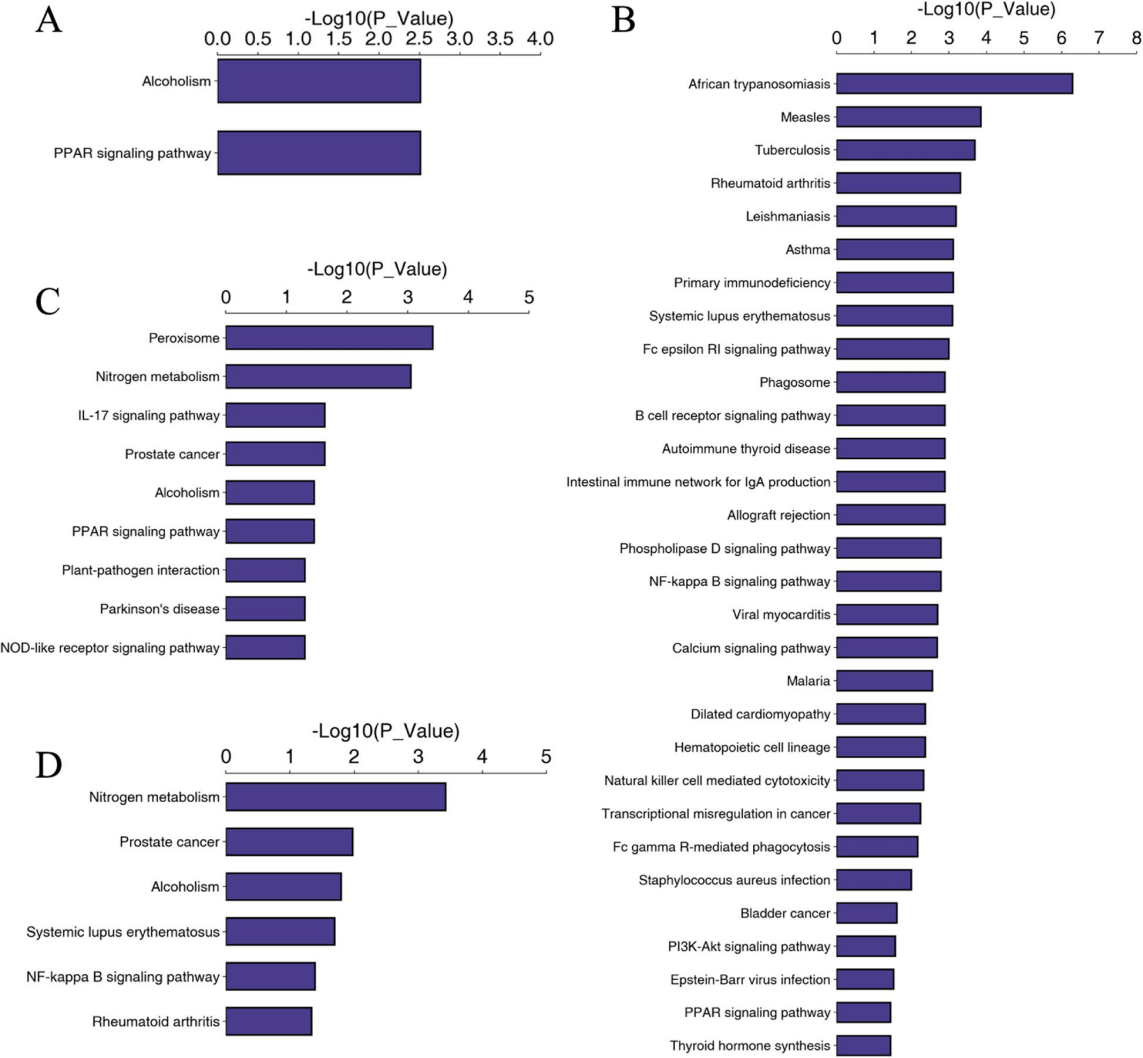
KEGG pathway enrichment analysis of the DEPs. The ordinate is the name of the pathway in which the DEPs are involved, and the abscissa is the size of *P* value involved in the pathway. **A** The KEGG pathway enrichment results of DEPs in AG vs. CTL. **B** The KEGG pathway enrichment results of DEPs in RG vs. CTL. **C** The KEGG pathway enrichment results of DEPs in AHU vs. CTL. **D** The KEGG pathway enrichment results of DEPs in AHU vs. AG

In order to classify the functional annotations of the identified proteins, pathway analysis of DEPs was mainly conducted by KEGG analysis (Tables S2, S3, S4 and S5). The top significant pathways in each comparison groups were displayed in Fig. 3. Although the KEGG analysis provides a large number of pathway information from each comparison groups, the most representative pathway was peroxisome proliferator activated receptor (PPAR) signaling pathways and alcoholism pathway, because these two pathways occurred frequently among four comparison groups. Moreover, interestingly, histone H2A and histone H2B proteins were seen to be involved in alcoholism pathways and these two proteins were significantly increased in AG, RG and AHU compared with CTL, but were significantly decreased in AHU compared with AG (Table 2). This result revealed that histone H2A and histone H2B proteins may be involved in the core mechanism of gout onset through alcoholism pathway.

**Table 2:** List of histone H2A and histone H2B in four comparison groups

**Accession**	**Description**	**AG/CTL**	***P*-value**	**AHU/CTL**	***P*-value**	**RG/CTL**	***P*-value**	**AG/AHU**	***P*-value**
A0A0U1RRH7	Histone H2A	4.132	0.005	1.553	0.030	1.437	0.003	0.348	0.009
B4DR52	Histone H2B	4.206	<0.001	1.951	0.025	1.913	0.022	0.455	0.004

The Table 1 shows the fold-change and its *P*-value of histone H2A and histone H2B in four comparison groups. Accession refers to protein numbers in the FASTA Database. Description refers to the name of protein

**Fig. 4:**
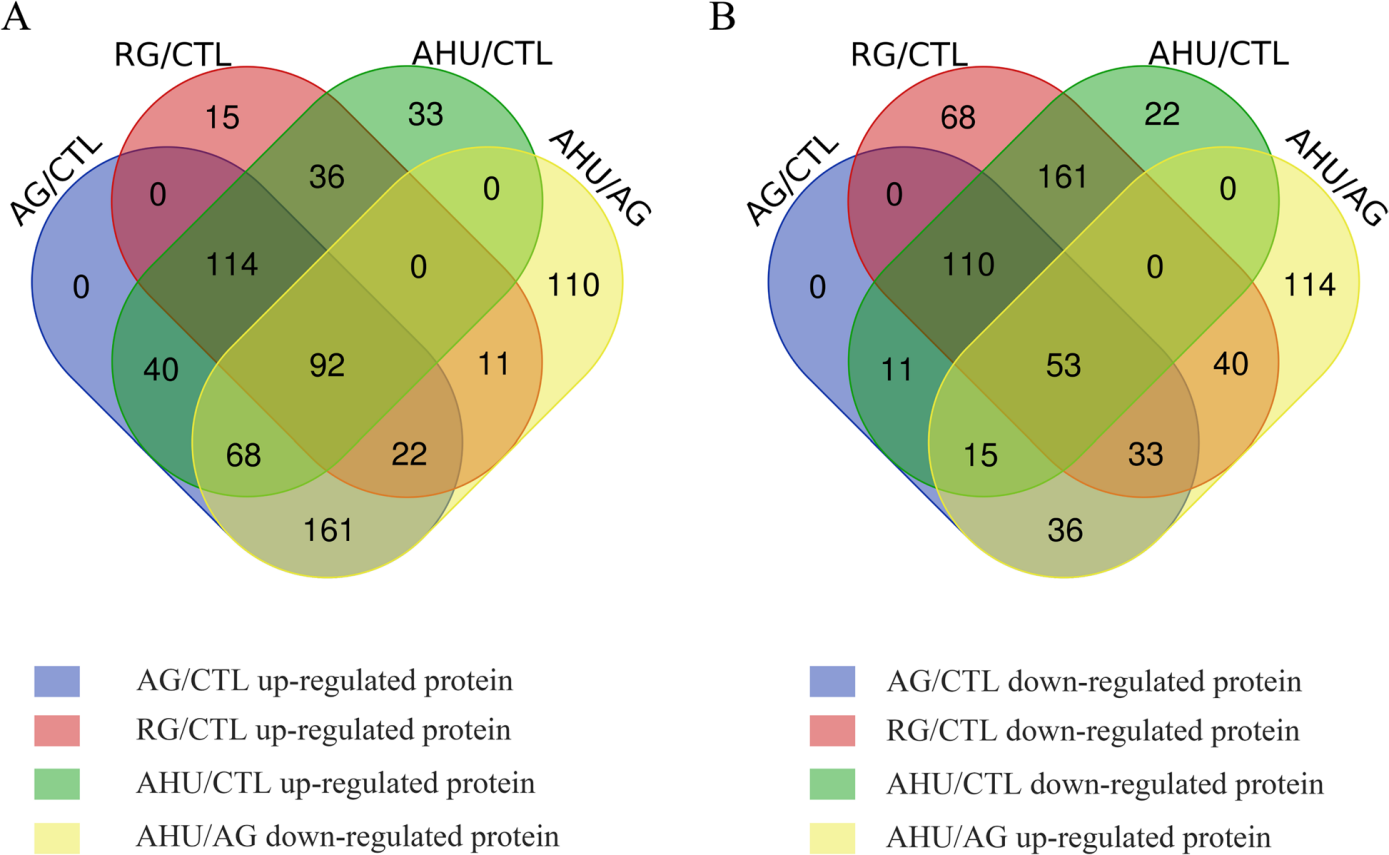
Venn diagram of unique and shared proteins of four comparison groups. The figure shows two different protein trends among four comparison groups to select proteins for further validated

Following this DEPs level trends, more proteins would be further validated by PRM analysis. Firstly, A venn diagram including the total DEPs from four comparison was generated to find the level trends we want. The detailed information of all proteins obtained from four comparison groups was presented in Fig. 4. In the venn diagram, 92 DEPs were shared in four comparison groups, which were significantly increased in AG, RG and AHU compared with CTL, but were significantly decreased in AHU compared with AG. Similarly, 53 DEPs were also shared in four comparison groups if the protein level trends become down-regulated in AG/CTL, RG/CTL, AHU/CTL, but up-regulated in AHU/AG. As shown in Fig. S2a, four groups were clearly separated from each other in PCA plot. Next, the supervised multivariate statistical method OPLS-DA was then employed to analyze each group (Fig. S2b ~ e). We found that the permutation test for OPLS-DA showed that the Q2 regression line had a negative intercept. Additionally, all R2 and Q2 values on the left were lower than the original points on the right, showing that the OPLS-DA model in the present study is valid (Fig. S2f ~ i). A total of 152 significantly DEPs (VIP > 1.0) were all successfully identified in the group of AG/CTL, RG/CTL and AHU/CTL (Fig. S2j). Then, 152 DEPs were further screened by a combination of veen result and protein sequence database searching based on DDA method. Finally, a list of 40 peptides was prepared for PRM validation (Table 3). Unfortunately, histone A and histone B was difficult to identified because its peptide spectrum matches (PSM) is lower from DDA database. (only the best scoring peptide to spectrum match for each LC/MS spectrum is considered as the potential peptide identification and is taken to the subsequent statistical validation).

**Table 3:** Selected 40 proteins to be verified by PRM

**Accession**	**Coverage**	**Unique peptide**	**AG/CTL**	***P* value**	**HUA/CTL**	***P* value**	**RG/CTL**	***P* value**	**AG/HUA**	***P* value**
Q14520	38	18	1.17	0.02	1.00	0.94	1.00	0.93	0.86	0.04
O95445	62	10	1.30	<0.01	1.15	0.01	1.29	0.10	0.99	0.91
P0DJI9	50	4	2.61	0.13	1.02	0.91	1.15	0.36	0.44	0.15
P23528	45	8	1.52	<0.01	1.37	0.17	1.42	0.17	0.93	0.70
P14780	23	14	1.50	<0.01	1.04	0.69	1.25	0.02	0.84	<0.01
P55056	30	5	2.17	<0.01	1.76	<0.01	1.90	<0.01	0.88	0.20
P02768	84	61	0.51	<0.01	0.43	<0.01	0.51	<0.01	0.99	0.84
P00734	56	35	1.04	0.13	1.00	0.96	0.97	0.29	0.93	0.02
P43652	45	25	0.90	<0.01	0.98	0.12	1.04	0.08	1.16	<0.01
P05160	49	29	1.15	0.01	1.02	0.49	1.00	0.96	0.87	<0.01
P05546	44	20	0.97	0.49	1.03	0.65	0.84	0.03	0.87	0.01
P06681	38	22	1.17	0.05	0.89	0.12	0.98	0.83	0.84	0.02
P02763	51	11	1.21	0.19	0.76	0.29	0.72	0.22	0.60	0.12
P19652	44	10	0.99	0.88	0.59	0.01	0.51	0.01	0.51	0.01
P05164	34	20	2.15	<0.01	0.95	0.63	1.18	0.30	0.55	0.01
P29401	32	17	0.91	0.10	0.72	0.02	0.96	0.28	1.06	0.34
Q9UHG3	34	14	1.22	0.07	1.08	0.35	1.14	0.42	0.93	0.61
P14625	22	16	1.00	0.98	0.83	0.02	0.79	<0.01	0.79	0.01
P02790	72	33	0.91	<0.01	0.98	0.15	0.92	0.22	1.01	0.92
P08697	49	22	0.93	0.30	0.95	0.33	0.94	0.27	1.01	0.82
Q96PD5	44	17	0.87	0.05	0.90	0.03	0.99	0.93	1.14	0.12
P04278	50	13	0.60	<0.01	0.70	0.01	0.72	0.03	1.20	0.23
P51884	38	10	0.79	<0.01	0.94	0.04	0.87	0.03	1.10	0.14
P35908	42	15	0.76	0.32	0.87	0.58	0.88	0.70	1.17	0.56
Q03591	28	1	0.97	0.73	0.99	0.87	0.99	0.81	1.01	0.86
P22352	29	6	0.83	0.02	0.88	0.02	0.87	0.07	1.05	0.43
Q14624	49	38	1.19	0.22	1.37	0.20	1.15	0.39	0.96	0.80
P05155	31	19	1.01	0.63	1.04	0.60	0.84	0.01	0.83	0.01
P07358	41	18	1.01	0.81	1.00	0.90	1.01	0.78	1.00	0.98
P07996	29	29	1.59	0.03	1.78	0.01	1.52	0.02	0.96	0.76
P02760	42	11	1.06	0.50	1.10	0.24	1.06	0.50	1.00	0.98
P02671	35	21	1.34	0.04	0.81	0.02	0.92	0.16	0.68	0.02
Q92954	20	23	1.33	0.02	1.18	0.01	1.13	0.04	0.85	0.10
O43866	47	17	1.66	0.02	0.92	0.47	1.12	0.09	0.68	0.05
P00915	57	10	1.06	0.63	1.42	0.22	2.19	0.02	2.06	0.03
Q04756	22	12	1.08	0.09	1.03	0.65	1.01	0.78	0.94	0.17
P00558	47	13	1.15	0.33	0.91	0.48	1.09	0.38	0.95	0.67
Q13201	16	14	1.20	0.08	1.10	0.34	1.17	0.19	0.97	0.76
O00187	17	9	1.18	0.06	0.97	0.70	1.09	0.23	0.93	0.01
P02741	23	5	1.73	0.11	0.71	0.32	1.35	0.41	0.78	0.41

The table shows the fold-change and its *P*-value of 40 selected proteins from iTRAQ data in four comparison groups. Accession refers to protein numbers in the FASTA Database. Coverage refers to the percentage of the protein sequence covered by identified peptides. Unique Peptides refers to the number of peptide sequences unique to a protein group

### PRM result

The PRM verified data were imported into skyline to check the peak shape of the target peptide segment and judge the spectral effect. The peak shape of some peptide segments was intact and the peak time was within the set retention time range, indicating the data quality was reliable (Supplementary Figure S1). Forty proteins related to gout process were found for PRM further analysis.

**Fig. 5:**
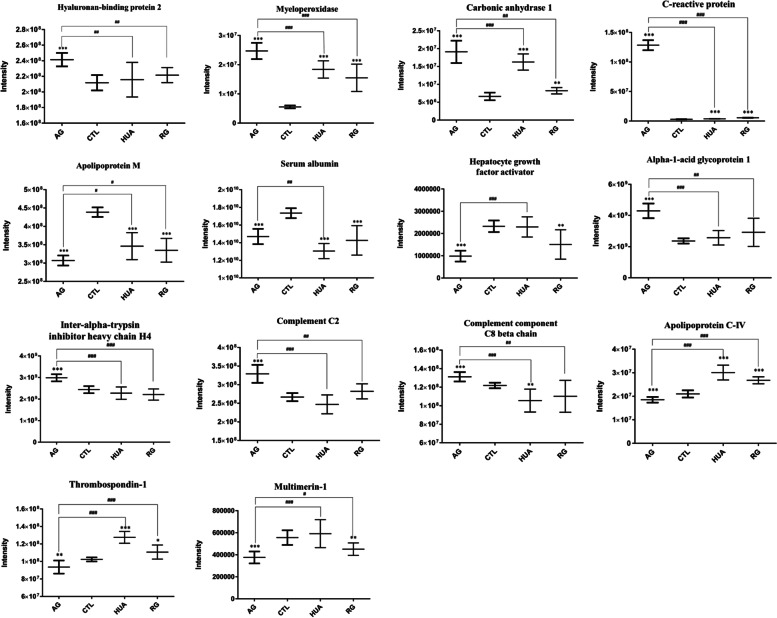
The comparison of protein expression by PRM. The ordinate is the group, and the abscissa is the intensity of protein level

PRM analysis revealed that 14 proteins were identified to predict gout process significantly. The results, as shown in Fig. 5, the level of four proteins (Hyaluronan-binding protein 2, Myeloperoxidase (MPO), Carbonic anhydrase 1 (CA1), C-reactive protein) were significantly increased in AG, RG and AHU compared with the healthy group. Interestingly, these four proteins were also expressed higher in AG than in RG and AHU. Similarly, the expression levels of Apolipoprotein M, Serum albumin (ALB) and Hepatocyte growth factor activator exhibited a significant reduction in AG, RG and AHU compared with the healthy group. And these three proteins were also expressed lower in AG than in RG and AHU. Alpha-1-acid glycoprotein 1 (ORM1), Inter-alpha-trypsin inhibitor heavy chain H4 (ITIH4) and Complement C2 presented no significant difference among healthy group, RG patients and AHU patients. However, in AG patients, the level of these two proteins were significantly lower than in the rest of three group. The levels of Complement component C8 beta chain (C8B) in AUR and AG patients were significantly lower than those of controls, which resulted in a significantly higher level in AG patients oppositely. More interestingly, the changes of Apolipoprotein C-IV and Thrombospondin-1 (THBS1) in RG and AHU patients were observed to increase compared with the healthy group but these two proteins displayed a significant reduction in AG patients compared with the healthy group. Finally, the level of Multimerin-1 (MMRN1) in AG patients was expressed lowest compared with the group of healthy control, RG and AHU.

**Fig. 6:**
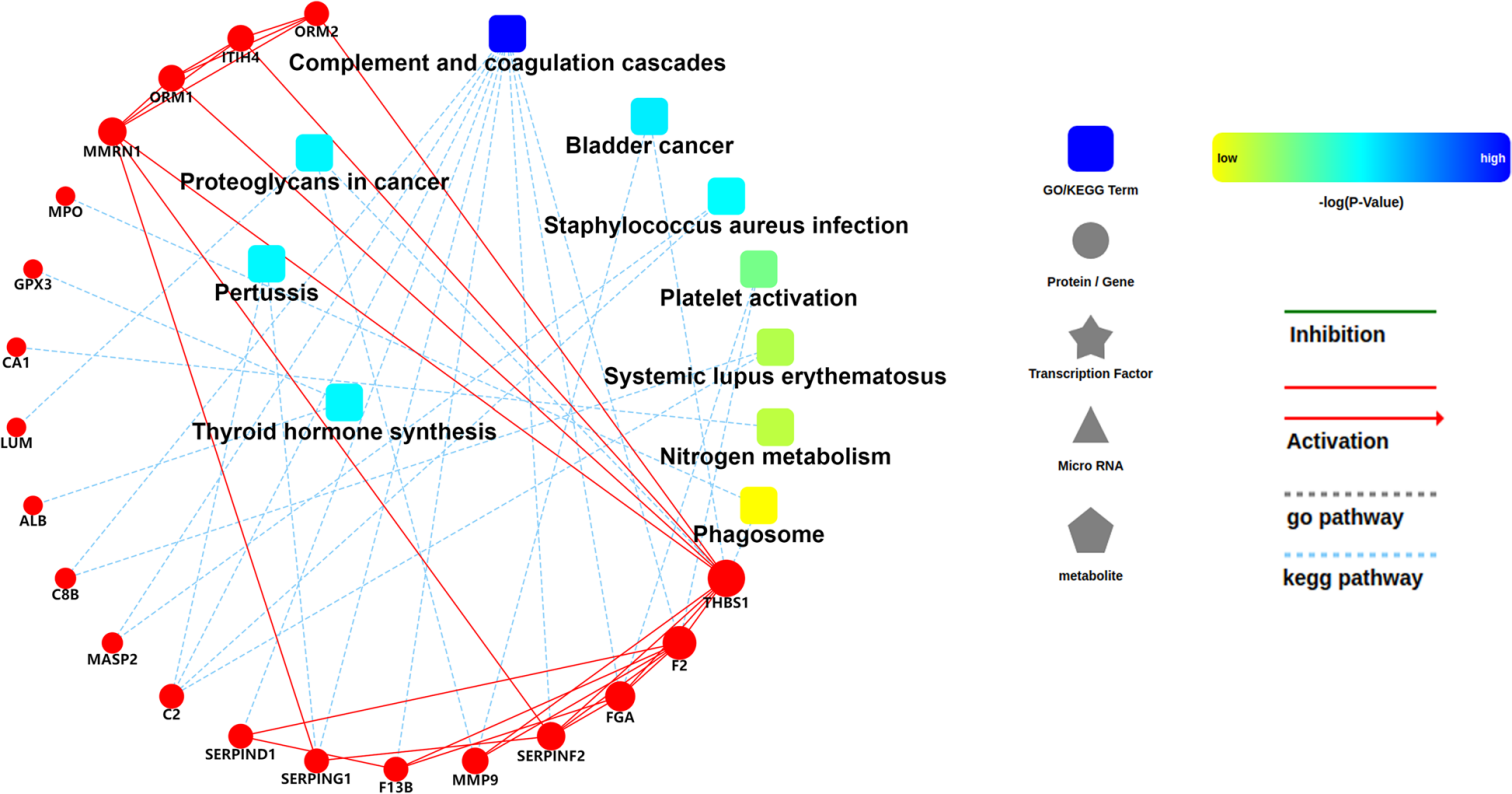
PPI network of 40 DEPs in each comparison groups. In this PPI network, circle nodes represent proteins and the size of nodes represents value of betweenness centrality corresponding to interconnectedness. Red solid lines represent the interactions between proteins; Square nodes represent GO/KEGG term, the color of nodes represents *P*-value and blue dashed lines represent statistically significant signaling pathways involved in DEPs

In order to reveal the function of proteins, 40 differential proteins related to gout process were selected for protein–protein interaction (PPI) network analysis. By comparing proteins to STRING, the results showed that known proteins, such as THBS1, F2, FGA, SERPINF2, ORM2, ITIH4, ORM1 and MMRN1, account for a large weight in the network (Fig. 6). However, combined with the PRM results, THBS1, ITIH4, ORM1, MMRN1, MPO, CA1, ALB, C8B and Complement C2 were significantly related to gout process. Of all proteins, THBS1 exhibited the strongest regulatory ability above all others and complement and coagulation cascades performed the strongest regulatory ability above all pathways due to its higher interconnectedness in the network. The THBS1 might be the key biomarker to maintain the balance and stability of the gout process.

## Discussion

In this study, we firstly use iTRAQ approaches in conjunction with PRM analysis to perform a comprehensive profile of the composition and differentiation of proteins among AG, RG, AHU and healthy control group. Eleven key proteins (histone H2A, histone H2B, THBS1, ITIH4, ORM1, MMRN1, MPO, CA1, ALB, C8B and C2) were detected among AG, RG, AHU and control group by combination of iTRAQ and PRM-based proteomics and bioinformatics analysis.

Among these proteins, histone H2A, histone H2B and THBS1 might be the strongest influential regulator to maintain the balance and stability of the gout process and complement and coagulation cascades is one of the main functional pathways in the mechanism of gout process. A growing number of studies have shown that histones can be released by neutrophil extracellular traps (NETs) formation [14, 15], which exhibit strong inflammatory activity both in vivo and in vitro. And evidences suggest that MSU crystals activate infiltrating neutrophils by inducing to form NETs [16]. Moreover, Neutrophils release myeloperoxidase (MPO) from their granules to form NETs in response to proinflammatory cytokines and MSU crystals which may also accelerate oxidative stress [17]. This significant correlation between the MPO and histone related to gout is similar with our established findings that MPO, histone H2A and histone H2B were significantly increased in AG, RG and AHU compared with the CTL. And we also discover that in RG and AHU group, MPO expressed lower than in AG group. These differences can be explained in part that the MPO becomes inactivated either by its substrate hydrogen peroxide or its product hypochlorous acid [18, 19]. Interestingly, thrombospondin-1 (THBS1) exhibited the strongest regulatory ability by interaction between other proteins including ITIH4, ORM1, ORM2, MMRN1, F2, FGA, SERPINF2, MMP9 (Fig. 6). Several studies stressed that elevated THBS1 is correlated with increased levels of proinflammatory cytokines in plasma of RA patients through TGF-β1/TSP-1 axis in vivo and in vitro [20, 21]. However, the findings of the current study do not support the previous research. We identified that THBS1 in RG and AHU patients were observed to increase compared with the healthy group but displayed a significant reduction in AG patients compared with the healthy group. A possible explanation for this might be that THBS1 is not directly involved in the course of gout. THBS1 is an adhesive glycoprotein that mediates cell-to-cell and cell-to-matrix interactions [22]. So THBS1 negatively regulates disease by acting indirectly on other gout related proteins. However, more research on this topic needs to be undertaken before the association between THBS1 and gout process is more clearly understood. Furthermore, evidences from PPI analysis indicated that complement component C8 beta chain (C8B) and complement C2 is involved in complement and coagulation cascades which plays a key role in the innate and adaptive immune response. Thereinto, C8B mediates the interaction of C8 with the C5b-7 complex to form membrane attack complex (MAC) [23]. As an intrinsic constituent of the classical activation pathway, complement C2 is involved in the formation of C3 convertase and C5 convertase and later components of the complement cascade further form the MAC [24]. Specifically, MSU crystals promote inflammation by providing a surface for cleavage of C5 and formation of MAC, culminating in secretion of cytokines and chemokines with a dramatic influx of neutrophils into the joint [25]. Further work needs to be done to establish how C8B and Complement C2 regulate gout process by complement and coagulation cascades.

In addition, several of the other proteins we identified have certain potential biomarker capabilities. Alpha-1-acid glycoprotein 1 (ORM1) and Inter-alpha-trypsin inhibitor heavy chain H4 (ITIH4) present same expression level trend in PRM result. ORM1 and ITIH4 are two remarkable acute-phase inflammatory response proteins [26, 27]. A survey conducted by Fourniera et al. have shown that expression of the ORM1 is controlled by cytokine network involving mainly interleukin-1β (IL-1β) [28]. Coincidentally, there is sufficient evidence supporting the IL-1 have key roles in initiation of acute gout flares and use of IL-1 inhibitor can shorten and prevent gout attack [29, 30]. However, the biological function of these two proteins in gout process remains unknown. More data are needed to assess the role of ITIH4 and ORM1 in the disease. The expression level trend of Carbonic anhydrase I (CA1) in this result is consistent with MPO in our present result. CA1 was usually described as a function of hydrating carbon dioxide reversibly [31]. However, its role in the pathogenesis of gout has not been discovered. Surveys such as that conducted by Zhang et al. have shown that over-expression of CA1 may exacerbate joint inflammation and tissue destruction [32]. This previous study indicates that CA1 may play an essential role during acute inflammation in gout. Clinically, carbonic anhydrase inhibitor can reduce the resorption of bicarbonate from the proximal tubule in the kidneys, which directly causes increasing in bicarbonate excretion, thus these drugs can be used to treat gout by alkalizing urine. Following this thought, we assume that carbonic anhydrase I may exacerbate gout by blocking the alkalinization of urine. Multimerin-1 is a factor V/Va-binding protein and may function as a carrier protein for platelet factor V which may perform platelet aggregation and clot formation. Some evidence suggests that platelet activation is exacerbated in gout, especially during gout flares [33]. Whereas, this published finding is contrary to our result which have suggested that multimerin-1 (MMRN1) in AG patients was expressed lowest compared with the group of healthy control, RG and AHU. The reason of this inconsistency requires further investigated. A number of previous contradictory studies suggest an interaction between MSU crystals and human serum albumin (ALB) in vitro with reference to the disease of gout [34]. Some argue that ALB inhibits MSU crystallization. Others propose a mechanism that ALB induces quicker precipitation in vitro of MSU and acts as a nucleator [35]. Our study indicates that serum albumin (ALB) exhibited a significant reduction in AG, RG and AHU compared with the healthy group and were also expressed lower in AG than in RG and AHU. The result observed in our investigation supports that ALB prevents initiation of acute gout flares. Considerably more work will need to be done to determine what role does ALB play in the pathogenesis of gout.

Parallel reaction monitoring (PRM), as a liquid chromatography-mass spectrometry (LC-MS)-based targeted protein quantification technique has been successfully utilized in the confirmation of relative abundance of proteins and their posttranslational modifications with its high resolution and high accuracy [10]. PRM is also expected to replace traditional validation methods such as western blot in the future. This is the first study to integrate this advanced approach intended to figure out potential candidate genes for novel biomarkers related to gout. However, with regard to the research methods, some limitations need to be acknowledged. Firstly, with a small sample size, selection bias must be exhibited, as the findings might not be transferable to clinical application. Secondly, clinical value of these new biomarkers cannot be obtained by mapping the ROC curve due to a small sample size. Therefore, large prospective cohort study could provide more definitive evidence to determine diagnostic and predictive value of these new gout related proteins.

## Conclusion

We investigated the application of iTRAQ and PRM based proteomics to explore key proteins alterations from AG, RG, AHU patients and healthy control. Our study revealed that these key proteins (histone H2A, histone H2B, THBS1, ITIH4, ORM1, MMRN1, MPO, CA1, ALB, C8B and C2) can be the potential marker to classify these four groups. Such complement and coagulation cascades may be one of the main functional pathways in the key mechanism of gout process. These data will hold promise for further clinical development of early prediction and diagnosis of gout.

## Supplementary Information

**Additional file 1: Table S1.** Details of differentially proteins. **Table S2.** The mapped signaling pathways of the differentially expressed proteins between AG and control. **Table S3.** The mapped signaling pathways of the differentially expressed proteins between RG and control. **Table S4.** The mapped signaling pathways of the differentially expressed proteins between AHU and control. **Table S5.** The mapped signaling pathways of the differentially expressed proteins between AG and AHU. **Table S6.** Sheet 1. Information about all candidate peptides of the protein to be validated. Sheet 2. A list of peptides from DDA analysis. **Table S7.** OLPS-DA VIP score more than 1. **Figure S1.** Example chromatogram of peptide segments. **Supplementary Figure S2.** Serum profles of DEPs for the healthy controls (CTL), the patients with acute gout (AG), remission of gout (RG) and asymptomatic hyperuricemia (AHU).

## Data Availability

Readers can access the data supporting the conclusions of the study by requesting them from the authors.

## References

[1] Dalbeth N, Choi HK, Joosten LAB, Khanna PP, Matsuo H, Perez-Ruiz F (2019). Gout. Nat Rev Dis Primers.

[2] Robinson PC (2018). Gout - an update of aetiology, genetics, co-morbidities and management. Maturitas.

[3] Kuo CF, Grainge MJ, Zhang W, Doherty M (2015). Global epidemiology of gout: prevalence, incidence and risk factors. Nat Rev Rheumatol.

[4] Liu R, Han C, Wu D, Xia X, Gu J, Guan H (2015). Prevalence of hyperuricemia and gout in mainland China from 2000 to 2014: a systematic review and meta-analysis. Biomed Res Int.

[5] Luo Y, Wang L, Liu XY, Chen X, Song YX, Li XH (2018). Plasma profiling of amino acids distinguishes acute gout from asymptomatic hyperuricemia. Amino Acids.

[6] Pernemalm M, Lehtiö J (2014). Mass spectrometry-based plasma proteomics: state of the art and future outlook. Expert Rev Proteom.

[7] Rauniyar N, Yates JR (2014). Isobaric labeling-based relative quantification in shotgun proteomics. J Proteome Res.

[8] Wang J, Gao L, Lee YM, Kalesh KA, Ong YS, Lim J (2016). Target identification of natural and traditional medicines with quantitative chemical proteomics approaches. Pharmacol Ther.

[9] Cehofski LJ, Honoré B, Vorum H (2017). A review: proteomics in retinal artery occlusion, retinal vein occlusion, diabetic retinopathy and acquired macular disorders. Int J Mol Sci.

[10] Vidova V, Spacil Z (2017). A review on mass spectrometry-based quantitative proteomics: targeted and data independent acquisition. Anal Chim Acta.

[11] Neogi T, Jansen TL, Dalbeth N, Fransen J, Schumacher HR, Berendsen D (2015). 2015 gout classification criteria: an American College of Rheumatology/European League Against Rheumatism collaborative initiative. Ann Rheum Dis.

[12] de Lautour H, Taylor WJ, Adebajo A, Alten R, Burgos-Vargas R, Chapman P (2016). Development of preliminary remission criteria for gout using Delphi and 1000Minds consensus exercises. Arthritis Care Res.

[13] MacLean B, Tomazela DM, Shulman N, Chambers M, Finney GL, Frewen B (2010). Skyline: an open source document editor for creating and analyzing targeted proteomics experiments. Bioinformatics.

[14] Chen R, Kang R, Fan XG, Tang D (2014). Release and activity of histone in diseases. Cell Death Dis.

[15] Franklin BS, Mangan MS, Latz E (2016). Crystal formation in inflammation. Annu Rev Immunol.

[16] Hahn J, Knopf J, Maueröder C, Kienhöfer D, Leppkes M, Herrmann M (2016). Neutrophils and neutrophil extracellular traps orchestrate initiation and resolution of inflammation. Clin Exp Rheumatol.

[17] Stamp LK, Turner R, Khalilova IS, Zhang M, Drake J, Forbes LV (2014). Myeloperoxidase and oxidation of uric acid in gout: implications for the clinical consequences of hyperuricaemia. Rheumatology (Oxford, England).

[18] Maitra D, Shaeib F, Abdulhamid I, Abdulridha RM, Saed GM, Diamond MP (2013). Myeloperoxidase acts as a source of free iron during steady-state catalysis by a feedback inhibitory pathway. Free Radic Biol Med.

[19] Paumann-Page M, Furtmüller PG, Hofbauer S, Paton LN, Obinger C, Kettle AJ (2013). Inactivation of human myeloperoxidase by hydrogen peroxide. Arch Biochem Biophys.

[20] Suzuki T, Iwamoto N, Yamasaki S, Nishino A, Nakashima Y, Horai Y, et al. Upregulation of thrombospondin 1 expression in synovial tissues and plasma of rheumatoid arthritis: role of transforming growth factor-β1 toward fibroblast-like synovial cells. J Rheumatol. 2015;42(6):943–7. 10.3899/jrheum.141292 25934826

[21] Ohyama K, Ueki Y, Kawakami A, Kishikawa N, Tamai M, Osaki M (2011). Immune complexome analysis of serum and its application in screening for immune complex antigens in rheumatoid arthritis. Clin Chem.

[22] Lawler J (1986). The structural and functional properties of thrombospondin. Blood.

[23] Rauniyar N (2015). Parallel reaction monitoring: a targeted experiment performed using high resolution and high mass accuracy mass spectrometry. Int J Mol Sci.

[24] Lintner KE, Wu YL, Yang Y, Spencer CH, Hauptmann G, Hebert LA (2016). Early components of the complement classical activation pathway in human systemic autoimmune diseases. Front Immunol.

[25] Cronstein BN, Terkeltaub R (2006). The inflammatory process of gout and its treatment. Arthritis Res Ther.

[26] Chen Y, Zeng C, Zeng H, Zhang R, Ye Z, Xing B (2015). Comparative serum proteome expression of the steroid-induced femoral head osteonecrosis in adults. Exp Ther Med.

[27] Sun H, Pan L, Jia H, Zhang Z, Gao M, Huang M (2018). Label-free quantitative proteomics identifies novel plasma biomarkers for distinguishing pulmonary tuberculosis and latent infection. Front Microbiol.

[28] Fournier T, Medjoubi NN, Porquet D (2000). Alpha-1-acid glycoprotein. Biochim Biophys Acta.

[29] So A, Dumusc A, Nasi S (2018). The role of IL-1 in gout: from bench to bedside. Rheumatology (Oxford, England).

[30] Szekanecz Z, Szamosi S, Kovács GE, Kocsis E, Benkő S (2019). The NLRP3 inflammasome - interleukin 1 pathway as a therapeutic target in gout. Arch Biochem Biophys.

[31] Supuran CT (2018). Carbonic anhydrase activators. Future Med Chem.

[32] Zheng Y, Wang L, Zhang W, Xu H, Chang X (2012). Transgenic mice over-expressing carbonic anhydrase I showed aggravated joint inflammation and tissue destruction. BMC Musculoskelet Disord.

[33] Conway R, Murphy CL, Madigan A, Kavanagh P, Geraghty L, Redmond N (2018). Increased platelet reactivity as measured by plasma glycoprotein VI in gout. Platelets.

[34] Perl-Treves D, Addadi L (1988). A structural approach to pathological crystallizations. Gout: the possible role of albumin in sodium urate crystallization. Proc R Soc Lond B Biol Sci.

[35] Kippen I, Klinenberg JR, Weinberger A, Wilcox WR (1974). Factors affecting urate solubility in vitro. Ann Rheum Dis.

