# Supplementary material for: Quantitative proteomics by iTRAQ-PRM based reveals the new characterization for gout
Source: Proteome Sci. 2021 Oct 11;19:12. doi: 10.1186/s12953-021-00180-0 (PMC8507311; doi:10.1186/s12953-021-00180-0)
Supplement: Supplementary file 1 — Additional file 1: Table S1. Details of differentially proteins. Table S2. The mapped signaling pathways of the differentially expressed proteins between AG and control. Table S3. The mapped signaling pathways of the differentially expressed proteins between RG and control. Table S4. The mapped signaling pathways of the differentially expressed proteins between AHU and control. Table S5. The mapped signaling pathways of the differentially expressed proteins between AG and AHU. Table S6. Sheet 1. Information about all candidate peptides of the protein to be validated. Sheet 2. A list of peptides from DDA analysis. Table S7. OLPS-DA VIP score more than 1. Figure S1. Example chromatogram of peptide segments. Supplementary Figure S2. Serum profles of DEPs for the healthy controls (CTL), the patients with acute gout (AG), remission of gout (RG) and asymptomatic hyperuricemia (AHU). [file 12953_2021_180_MOESM1_ESM.zip › Supplemental Figure.doc]

**Supplementary Data**

**Supplementary Figure S1 example chromatogram of peptide segments.** The PRM-validated data was imported into SKYLINE, the peak shape of the target peptide segment was checked, and effect of the spectrogram was determined. If the peak shape is intact and the peak time is within the set retention time range, the data quality is reliable. The figure displays one of the all proteins (O00187). The abscissa represents the retention time. The ordinate is a representation of the intensity of peptides.

**Supplementary Figure S2 Serum profles of DEPs for the healthy controls (CTL), the patients with acute gout (AG), remission of gout (RG) and asymptomatic hyperuricemia (AHU).** (A) 2-Dimensional PCA score plots show visual separation of four groups. R2X=0.287, Q2=0.225. (B) 2-dimensional OPLS-DA score plot highlights the difference between the AG patients (purple spheres) and CTL (blue spheres). R2X=0.782, R2Y=0.864, Q2=0.308. (C) 2-dimensional OPLS-DA score plot highlights the difference between the RG patients (yellow spheres) and controls (blue spheres). R2X=0.765, R2Y=0.791, Q2=0.382. (D) 2-dimensional OPLS-DA score plot highlights the difference between the AHU patients (red spheres) and controls (blue spheres). R2X=0.805, R2 Y=0.757, Q2=0.482. (E) 2-dimensional OPLS-DA score plot highlights the difference between the AHU patients (red spheres) and AG patients (purple spheres). R2X=0.514, R2Y=0.977, Q2=0.202. The permutations plots for the CTL VS AG (F), CTL VS RG (G), CTL VS AHU (H) and AG VS AHU (I) all show the OPLS-DA models used above are valid. The green dots represent R2 while blue dots represent Q2. The dash lines represent the corresponding regression lines consistent with the dots color. The number of permutations for each plot was 200. (J) A Venn diagram was applied to illustrate that candidate DEPs with VIP scores greater than 1 in all three comparison groups.
